# Detecting Comparative Features of Comprehensive Geriatric Assessment through the International Classification of Functioning, Disability, and Health Linkage: A Web-Based Survey

**DOI:** 10.3390/jcm12154917

**Published:** 2023-07-26

**Authors:** Naoki Tomita, Yuki Ohashi, Aiko Ishiki, Akiko Ozaki, Mitsuyuki Nakao, Satoru Ebihara, Yasuyuki Taki

**Affiliations:** 1 Department of Geriatric Medicine and Neuroimaging, Tohoku University Hospital, Sendai 980-8575, Miyagi, Japan; 2 Department of Aging Research and Geriatric Medicine, Division of Brain Science, Institute of Development, Aging, and Cancer (IDAC), Tohoku University, Sendai 980-8575, Miyagi, Japan; 3 Nursing Department, Rakuwakai Otowa Rehabilitation Hospital, Kyoto City 607-8062, Kyoto Prefecture, Japan; 4 Department of Community Medicine, Tohoku Medical and Pharmaceutical University, Sendai 981-8558, Miyagi, Japan; 5 Department of Gerontological and Home Healthcare Nursing, Division of Health Sciences, Graduate School of Medicine, Tohoku University, Sendai 980-8575, Miyagi, Japan; 6 Laboratory of Biomodeling, Department of Applied Information Sciences, Graduate School of Information Sciences, Tohoku University, Sendai 980-8579, Miyagi, Japan; 7 Unprecedented-Scale Data Analytics Center, Tohoku University, Sendai 980-8579, Miyagi, Japan; 8 Department of Internal Medicine and Rehabilitation Science, Graduate School of Medicine, Tohoku University, Sendai 980-857, Miyagi, Japan; satoru.ebihara.c4@tohoku.ac.jp

**Keywords:** comprehensive geriatric assessment, environment, functional ability, geriatric syndrome, international classification of functioning, disability, and health, interoception, intrinsic capacity, proactiveness, quality of life

## Abstract

Multidimensional assessments are important in evaluating the overall health of older adults. The comprehensive geriatric assessment (CGA) is a representative framework; however, the burden associated with the CGA has led to the development of simplified multidimensional tools. Comparing these tools to the CGA can help utilize them effectively. However, a direct comparison is challenging owing to the conceptual nature of the CGA. In this study, we conducted a web-based survey to identify essential CGA components by linking International Classification of Functioning, Disability, and Health (ICF) category level 2 items and “not defined/not covered” (nd/nc) items. Healthcare professionals and individuals aged >65 years participated in a two-stage Delphi study. In total, 182 respondents (7 geriatricians, 22 nurses, 20 therapists, and 4 case managers) completed the survey. Sixty-one essential components for CGA were identified, including 55 ICF categories. Additionally, personal factors (i.e., proactiveness) and nd/nc items (i.e., subjective perceptions) were aggregated. The results suggest that the CGA includes objective conditions of intrinsic capacity, functional ability, and environment as well as subjective perceptions and proactiveness toward those conditions. Thus, CGA is not merely expected to assess geriatric syndrome but also to estimate broader concepts, such as interoception, resilience, and quality of life.

## 1. Introduction

Old age increases the risk of developing multimorbidity, disabilities, and multifactorial symptoms. These factors, including diverse social backgrounds, lead to fragmented healthcare [1,2,3]. Therefore, in addition to focusing on specific illnesses and symptoms, simultaneously screening overall health is essential for older adults. “Multidimensional”, “comprehensive”, and “holistic” are common terms describing overall health screening. The term “multidimensional” represents more than two components, whereas “holistic” describes all components of a person’s health. The definition of “comprehensive” remains somewhat more ambiguous, as its constructs and components vary depending on the objectives.

As the goal of geriatric medicine is broadly stated as improving the quality of life (QOL) [1], it is natural to include the estimation of QOL in geriatric assessment. In contrast, more practical evaluations are required for geriatric assessment, such as body functions, activities of daily living (ADLs), degree of independence, risk of disability, geriatric syndromes, and living conditions.

In geriatric medicine, the “comprehensive geriatric assessment (CGA)” has been a well-known overall health evaluation method worldwide since the 1930s [4]. The objective of CGA has been addressed as estimating the QOL (referred to as the “classical CGA”) [1]. However, QOL is not sufficiently practical as a useful indicator in clinical practice [5,6,7]. Additional clarification is required to perform the classical CGA.

**Figure 1:**
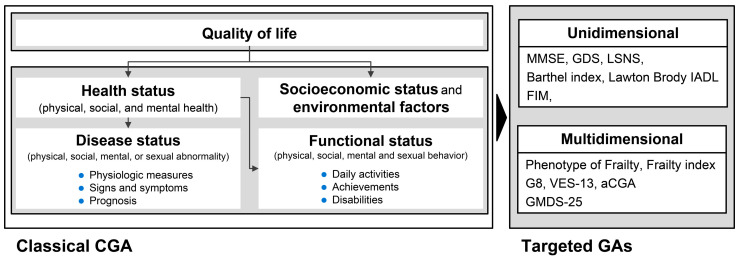
Classical CGA components and targeted geriatric assessments. The conceptual components of the classical CGA (left side of the figure) are shown in reference [1]. CGA, comprehensive geriatric assessment; GAs, geriatric assessments.

In addition, as performing the classical CGA is labor-intensive, the purpose of evaluation needs to be more narrowed for its effective use. In fact, recently developed geriatric assessment (GA) tools aim at a more specific target to ensure item specificity and the feasibility of evaluation in the actual clinical field (referred to as “Targeted” GAs). Targeted GAs include assessing elemental functions or conditions (“Unidimensional” GAs) and multidimensional GAs. A multidimensional assessment for older adults includes the classical CGA and targeted, multidimensional GAs (Figure 1).

Effective and feasible medical practice can be achieved using the best-fit assessment. Even in geriatric medicine, the classical CGA is not always the best-fit assessment. Recently, the importance of evaluating frailty has been emphasized for older adults [2]. It has even been stated that broadly assessing frailty-related conditions is consistent with the classical CGA, though frailty assessments have not been proven to be concordant with the classical CGA. In contrast, multidimensional assessments are indispensable even for older adults in specific fields of medicine. In fact, quite a few targeted GAs have been developed in many medical areas other than geriatric medicine.

Comparison with the classical CGA helps capture the characteristics of each multidimensional assessment. However, as the specific components of the classical CGA are not clearly defined, it is difficult to directly compare the characteristics of multidimensional assessments to the classical CGA by actual items. To consider the differences between the classical CGA and other multidimensional assessments, the components of the classical CGA must be clarified using definite and universal items.

In 2001, the World Health Organization (WHO) devised the International Classification of Functioning, Disability, and Health (ICF) to provide a unified and standard language and framework for describing health and health-related states, prompting the systematic coding of health components [8]. The ICF classification is hierarchical (Figure S1). The items are divided into (I) functioning/disability and (II) contextual factors. Part I consists of body function and structure and activities and participation. Part II consists of environmental factors and personal factors (pfs). Except for pfs, all components are grouped into chapters and subdivided into categories, including second, third, and fourth levels, ensuring a common classification framework. As the ICF is complex for daily use, specific ICF code sets or ICF Core Sets exist for clinicians and other professionals for predefined purposes [9,10,11,12,13,14].

ICF linkage has been proposed using the second-level ICF category codes and implementation rules for existing measurement and evaluation methods [15,16]. CGA tools comprise items for combining geriatric syndromes and conditions, abstract concepts, and other assessment tools, resulting in redundancy or ambiguity. Using the ICF linkage system to form the classical CGA components agreed upon and comparing them with components of existing multidimensional geriatric assessment tools will clarify the characteristics of CGA.

The primary objective of the present study was to detect the essential components of the classical CGA by the ICF linkage to clarify the difference between the classical CGA and frailty assessments. Moreover, we aimed to compare the classical CGA components with other targeted GAs for their effectiveness. Finally, we discussed how the components of multidimensional assessment should be expressed by considering differences in opinions for inclusion between healthcare professionals and non-professional older individuals.

## 2. Materials and Methods

### 2.1. Study Design

We conducted a web-based survey on which items should be included in the classical CGA using two iterations of the Delphi technique, a widely accepted method for gathering and consolidating opinions among respondents without face-to-face discussions [17].

Healthcare professionals included geriatricians, nurses, therapists, and case managers. Survey invitations were sent out in March 2018 to all geriatricians and physiatrists affiliated with teaching hospitals and certified by the Japan Geriatric Society. Nurses, physical and occupational therapists (PTs and OTs, respectively), and case managers were selected using convenience sampling and were considered eligible for inclusion if they engaged in daily geriatric care.

We recruited older non-professionals: (1) who were registered respondents of a survey company; (2) who were caregivers for older adults belonging to the Alzheimer’s Association of Japan; and (3) through a published bulletin invitation. Individuals aged >65 or <65 years who provided care to older individuals were considered eligible.

### 2.2. Survey Questionnaire Details

The core members discussed the expression and type of questionnaire suitable for both professional and non-professional respondents. Instead of mentioning the CGA directly, simpler, clear terms should be used to explain the whole survey. By referring to the description of the classical CGA in textbooks [1,4], we decided to use the following explanation: “The present survey aims to clarify what should be measured and assessed for understanding the overall health profile of older people”.

The structure of the present survey questionnaire is described in Table 1. We first showed a list of geriatric syndromes and conditions often mentioned regarding health in old age. The list was provided to professionals and non-professional respondents who were asked to choose items from the list.

For the main part of the survey, we asked respondents to select all ICF categories that they think are essential for understanding the overall health profile, namely the classical CGA (Table 1 No. B1–B3), except for pfs, which have no further subdivided categories.

Overall, answer options for check-all-that-apply questions for B1–B3 were derived from the corresponding part of ICF second-category items. Response options for A1 and B4 were selected through a discussion between core members. For the response options for B4, we selected items that are often exemplified for pfs. For A1, we selected geriatric syndromes or conditions including frailty and related conditions that have little overlap with the physical aspects of frailty (Table 2).

We attached a representative illustration to each category to help respondents understand what the item represents. A scholar developed these illustrations, available online for understanding the ICF categories (ICF illustration library. URL: http://www.icfillustration.com/icfil_jpn/b/b.html, accessed on 9 May 2023). We obtained permission to use illustrations from the library for our survey.

The healthcare professionals provided feedback for all second-level ICF categories. In contrast, non-professional respondents were not offered questions concerning body function and structure as they lacked sufficient medical knowledge (Table 1, No. B3).

We also asked respondents to describe items they think should be included in the CGA but cannot be found in the ICF items we prepared. By consolidating these freely expressed answers, we aimed to consider CGA components that belong to “not defined/not covered (nd/nc) items” in ICF linkage rules (Table 1, No. A1, B1–B4).

The ICF is a universally accepted reference framework for comparing health information. It has also been used to link data obtained from open-ended questions. The refined ICF linking rules [16] are summarized in Table S1. Examples of ICF linking are presented in Table S2.

It is not always feasible to link the information to an ICF category, as some information is beyond the scope of the ICF or too specific to be covered within the ICF. We also present the ICF linking decision tree described previously [16] (Figure S2) for better understanding of the classification of the free answers derived from open-ended questions.

In the present survey, ICF second-category items were directly selected by the respondents for each component. After tallying, items were then sorted out in the predetermined manner described in Section 2.4. For information not contained in those ICF items, which were freely described in each open-ended questions, the core members first summarized the responses and, then, classified them by the ICF linking rules.

### 2.3. Comparison with Other Multidimensional Geriatric Assessments

We compared the second-level categories of the CGA core set with the comprehensive and brief Geriatric ICF Core Sets for post-acute rehabilitation facilities [10,11,12,13] and community-dwelling older adults [14].

We also compared the classical CGA with commonly used assessment tools for evaluating frailty [18], including the 70-item Frailty Index, Groningen Frailty Indicator (GFI), Frailty Index-CGA, the Kihon Checklist based on the accumulated deficit model [19,20], and Frailty Phenotype Cardiovascular Health Study Criteria [21]. The CGA core set was compared with GA tools used in oncology, including the Geriatric 8 (G8), Vulnerable Elders 13 Survey (VES-13), and abbreviated CGA (aCGA) [22].

### 2.4. Statistical Analysis

We selected the top 20% of items from each ICF category to identify the classical CGA components. The percentage differences between the responses of professionals and non-professional older adults were examined to consider whether any missing components exist. We used Microsoft Excel 2019 (Microsoft Corp., Redmond, WA, USA) to analyze and tabulate the baseline responses and percentages. To ensure reproducibility, accuracy was confirmed with SAS version 9.3 (SAS Institute Inc., Cary, NC, USA).

## 3. Results

### 3.1. Characteristics of the Respondents

**Figure 2:**
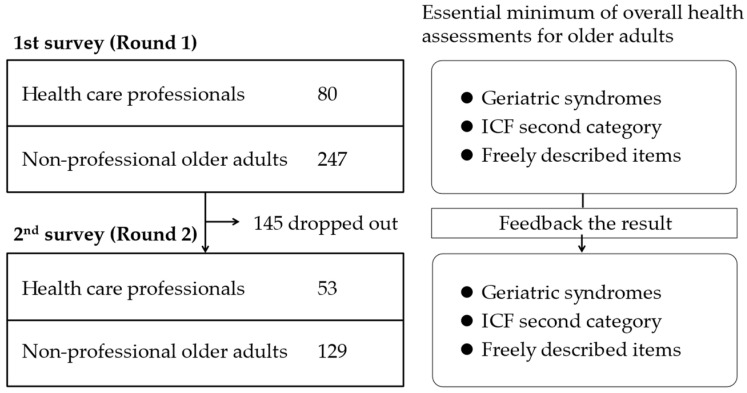
Flow chart of the Delphi process for the Internet survey. ICF, International Classification of Functioning, Disability, and Health.

We sent 155 survey invitation letters to certified geriatricians. Home health care nurses and case managers were invited to participate in this study by an administrator of their home care organization with the target of 30 respondents for each group. PTs and OTs were recruited, with the target of collecting 30 respondents for each, by inviting researchers in the field of rehabilitation. They participated voluntarily. For non-professional older adults, an advertisement was placed with the goal of collecting 300 respondents within the survey period. The actual number of respondents that agreed to participate was 327 in total. The abstract survey flow and the number of respondents are described in Figure 2.

Of the 327 individuals who completed the first round, 48.6% were women, 73 (22.3%) were medical professionals, 7 (2.1%) were care workers, and 247 (75.5%) were non-professionals. The professionals were significantly younger than the non-professionals (44.1 ± 11.3 vs. 69.4 ± 5.3 years). Of the 182 individuals who completed the second round, 50.0% were women, 49 (26.9%) were medical professionals, 4 (2.2%) were care workers, and 129 (70.9%) were non-professionals. The professionals were significantly younger than the non-professionals (43.1 ± 10.4 vs. 69.2 ± 3.6 years). The demographic characteristics of the respondents for the two-round Delphi process are described in Table 3.

### 3.2. Aggregated Items of the Classical CGA Components

Finally, 62 items were selected through the Delphi process and considered as the classical CGA components. These items comprise 52 (96.5%) second-level ICF categories, four pfs, and six nd/nc items.

#### 3.2.1. Categories Selected from the ICF Components

Fifty-five items were chosen from the ICF components, composed of 52 second-level ICF categories (22 (40.0%) from body function, 2 (3.6%) from body structure, 16 (29.1%) from activities and participation12 twelve (21.8%) from environmental factors), and 3 (5.5%) from pfs (Table 4).

#### 3.2.2. Open-Ended Question Responses for Inclusion in the CGA (Table 3)

The open-ended question responses of non-professionals comprised items covered by ICF, primarily in the body function (b) and body structure (s) categories. To avoid redundancy, responses that cannot be assigned to the ICF components, except for pfs, are presented in Table 5.

According to the 2016 version of the ICF Linking Rules [16], the open responses were categorized into one pf and six nd/nc categories, including not defined health condition (nd-hc), not defined disease (nd-dis), not covered (nc), not covered health condition (nc-hc), and not covered QOL (nc-QOL). Responses related to QOL (nc-QOL) were the most frequently answered. While the experts’ answers were conceptual, non-experts responded with more direct, concrete answers.

### 3.3. Common Geriatric Concepts Selected for Inclusion in the CGA (Table 6)

Social support and participation were unanimously recommended for inclusion by both groups. The medical staff favored the inclusion of social support (95.9%) over social participation (91.8%), whereas case managers and non-professionals showed no preference between the two parameters. The medical staff (71.4%) preferred the inclusion of frailty more than the non-medical groups (case manager, 25%; non-professionals, 20.2%) did.

### 3.4. Comparison of the Classical CGA Components with Existing Multidimensional Assessment Tools (Figure 2)

**Figure 3:**
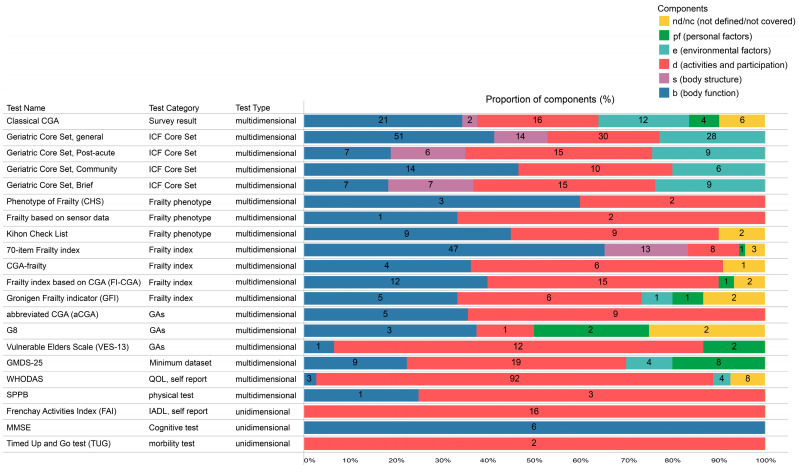
Comparison between the composition of the classical CGA and that of other geriatric assessments. ≠ The compositions of geriatric ICF Core Sets were derived from references [7,8,9,10,11]. WHODAS is derived from reference [21]. Others are derived from reference [15].

Through previous studies and reviews, we compared the classical CGA’s composition to other multidimensional geriatric assessments already linked to the ICF [18,23,24] (Figure 3).

#### 3.4.1. Comparison with the Geriatric ICF Core Set

The classical CGA components (*n* = 61) feature more second-level ICF categories than the brief ICF Core Sets (*n* = 38, 30) but have fewer features than the comprehensive ICF Core Set (*n* = 123). All core sets preferentially utilized items from the body function/structure (36.8–48.3%) and the activities and participation (29–39.4%) categories. Environmental factors (20.7–23.6%) were the least preferred. Only the classical CGA included pfs and nd/nc items.

#### 3.4.2. Comparison with Frailty Criteria

The frailty phenotype model comprises body function and structure criteria, whereas the deficit accumulation model comprises additional variable components. The classical CGA contains six components, whereas the 70-item Frailty Index and GFI, which assess the most variable component, have five domains. Further, compared with the classical CGA, most instruments do not cover the spectrum of the ICF components.

#### 3.4.3. Comparison with GA Tools for Clinical Oncology Criteria

The G8, VES-13, and aCGA indices are used in clinical oncology to evaluate body function, activities, and participation categories. The G8 and VES-13 indices include personal factors; however, only the G8 index includes nd/nc categories.

#### 3.4.4. Comparison of Not Defined/Not Covered (nd/nc) Items

For the nc-QOL of the classical CGA, the conceptual answers of experts and concrete answers of non-experts (Table 3) were summarized as “self-perception”. The nc-hc and a part of nd-dis of the classical CGA were summarized as “Geriatric syndrome/condition”. These summarizations were decided through a discussion between the core members, as it was necessary to make the comparison easier to understand.

Among the open responses without second-level ICF category codes, subcategories of nd/nc varied most widely in the classical CGA (Table 7).

## 4. Discussion

This study aimed to clarify the characteristics of multidimensional geriatric assessments, including the classical CGA and currently available multidimensional tools for older adults (ICF geriatric Core Set, Frailty index, and other frequently used practical tools), by linking these assessments to the ICF. We assumed the classical CGA as an overall health assessment for older adults. This inclusive assumption seems reasonable, as the term “CGA” varies widely and is used in various settings for various cases of individuals, including non-frail older adults [24]. In fact, multiple tools named “CGA” can be found in Figure 3. It would be useful to detect the comparative features of the classical CGA and other multidimensional assessments by the inclusive definition of the CGA.

**Figure 4:**
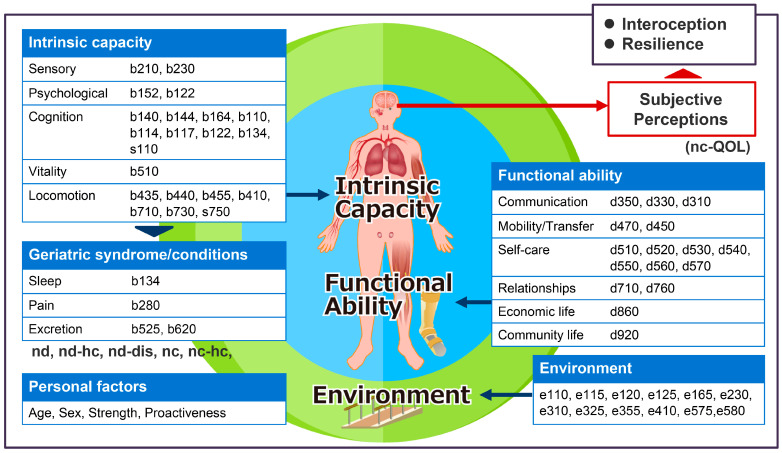
The presumed components of the classical CGA expressed by the CGA. CGA, comprehensive geriatric assessment; ICF, International Classification of Functioning, Disability, and Health; nc, not covered; nc-hc, not covered health condition; nc-QOL, not covered quality of life; nd, not defined; nd-dis, not defined disease; nd-hc, not defined health condition.

One major finding of the present research is that the classical CGA was thought to include all ICF components with some items without an ICF code, including proactiveness and subjective perceptions (Figure 4).

The goal of healthy aging is defined as helping people develop and maintain the functional ability that enables well-being [3]. Functional ability is defined as the “health-related attributes that enable people to be and to do what they have reason to value”. Functional ability consists of the intrinsic capacity of the individual, the environment of the individual, and the interactions between them. Intrinsic capacity is “the composite of all the physical and mental capacities that an individual can draw on” [3]. In 2019, the WHO released integrated care for older people guidelines for person-centered assessment and pathways in primary care to put these concepts into practice [25]. In the guideline’s handbook, the actual domains of intrinsic capacity are indicated as cognitive decline, limited mobility, malnutrition, visual impairment, hearing loss, and depressive symptoms. Our aggregated items of the classical CGA fit the categories very well. The selected sets of ICF items could be classified into intrinsic capacity with related symptoms, functional ability, and environment. The presumed components of the selected items are in good accordance with the already proposed configurations of ICOPE.

For those items without an ICF code, proactiveness and subjective perception would be characteristic results when considering the potential purpose of the CGA other than QOL. Comparing subjective perceptions with objective information would lead to interoception. Interoception refers to the sensation of physiological states inside the body, and its accuracy is known to decrease with age [26]. It could be practical and useful, as behaviors are known to be influenced by interoception. Proactiveness is considered to be a component of resilience [27], which may indicate that resilience is potentially thought of as a necessary concept for total health assessment.

The aggregated set of items for the classical CGA seems feasible (Table 2 and Table 3), as the number of items is more precise than brief geriatric screening tools [13] and more compact than the full Geriatric ICF Core Set [12]. The CGA has long been included in the core medical education curriculum. However, its components and constructs have not been sufficiently clarified. Instead of specifying the items of the classical CGA, it has been dealt with by narrowing down the evaluation target to practical concepts and measures (such as assessing frailty instead of overall health). We clarified the components of the classical CGA without limiting the original target of the CGA. We laid the groundwork for discussing how we achieve the overall health assessment of older adults.

Another finding concluded from the result of the A1 questionnaire (selection of geriatric syndromes and conditions) is that expressing the components of multidimensional geriatric assessments merely by the combination of geriatric syndromes or conditions would be redundant. A vast difference between professional and non-professional respondents existed in the proportion of frailty (Table 4); however, such difference was not observed in the proportion of the selected ICF category. We should be aware that the comparative characteristics of a multidimensional assessment would be redundant if explained solely by the combination of abstract concepts (such as “frailty plus QOL”) or the assessment tools (such as “Kihon Check List + SF-12”), as some of the subitems may duplicate, leading to redundancy. In addition, variation in evaluation items of the classical CGA would be minimized by using widely agreed upon as the smallest health component instead of merely expressing it via abstract concepts. The ICF is a classification of health and health-related domains and a practical tool to compare health information [8,15,16].

While the open responses of experts related to QOL (nc-QOL) were conceptual, non-experts responded with more direct concrete answers (Table 3). Non-professional respondents mainly answered subjective perceptions. Even if accurately measuring subjective perceptions is burdensome, especially in older adults, experts should be cautious not to leave this behind when assessing health. Measuring and estimating the accuracy of subjective perception is an issue for future research.

Our study’s results will significantly contribute to the proper use and combination of multidimensional geriatric assessments, which may lead to the improved utilization of digital health. It is becoming more feasible to perform multidimensional evaluations for wellness tracking and disease diagnosis, prevention, and management owing to the technological development of digital devices, such as sensors, mobile apps, social media, and location-tracking technology. In addition, the importance of digital health management is gaining momentum because of the COVID-19 pandemic. Digital health assessment is now moving from assessing a single disease or disability to assessing overall health [28]. Furthermore, various attempts have been made to evaluate wellness by comprehensively combining these technologies. However, various multidimensional geriatric assessments exist, from conceptual frameworks (such as the CGA) to practical tools (such as the ICF geriatric Core Sets).

### Limitations

This study had some limitations. First, in this study, iterations were limited to two to minimize the burden on older respondents, despite typical Delphi methods requiring three or more iterations for aggregation. The reduction helped maintain the number and quality of valid responses.

Second, an issue remains with the ICF link itself as a standardized method for converting health information into a universal health language. The ICF link clarifies the components of a concept, but by itself, it does not give a specific method of how it should be evaluated.

Third, the consensus of the present survey is limited to the Japanese population. We hope this survey’s results will lead to additional surveys in other countries. Comparing consensus internationally and determining if any differences arise in judging the comprehensiveness of the health assessment for older adults is valuable.

As therapists utilize the ICF in clinical practice, we intended to include speech therapists (STs), PTs, and OTs. However, we did not have any STs as respondents.

We sent an invitation to the national organization for geriatricians (the Japan Geriatric Association). Despite our efforts to streamline and simplify the questionnaire, the number of geriatricians who responded was limited, possibly owing to responder fatigue. We also recruited older respondents from three sources; most non-professionals were from the survey company. Caution must be exercised when generalizing their results.

We initially aimed to establish consensus using specific items in the second category of the ICF. However, the number and composition of respondents did not reach the ideal level. The resultant list of the ICF items or presumed components and constructs of the classical CGA should be treated as a reference for utilizing the list as a guide for developing clinical tools of the classical CGA, not as strict conditions.

## 5. Conclusions

The aggregated opinions of experts and older individuals identified 61 ICF second-level categories and six additional items, including subjective perception and proactiveness for the classical CGA components. The results suggest that the CGA includes not only objective conditions of intrinsic capacity, functional ability, and environment but also subjective perceptions and proactiveness toward these conditions. Thus, the CGA is expected to assess geriatric syndrome and estimate broader concepts, such as interoception, resilience, and QOL.

## Figures and Tables

**Table 1:** The content of the survey questionnaire.

No.	Questions Choose/Note Items for Assessing the Overall Health Profile	Response Methods
A1	List of geriatric problems or concepts * (Geriatric syndromes/Geriatric conditions)	Check-all-that-apply
	Note if you think of any other items regarding GS	Open-ended
B1	Activities and participation (d; ICF second category)	Check-all-that-apply
	Note if you think of any other items regarding d	Open-ended
B2	Environmental factors (e; ICF second category)	Check-all-that-apply
	Note if you think of any other items regarding e	Open-ended
B3	Body functions and structure (b, s; ICF second category) **	Check-all-that-apply
	Note if you think of any other items regarding b and s	Open-ended
B4	List of pfs	Check-all-that-apply
	Note if you think of any other items regarding pf	Open-ended

* See Table S2; ** B3 was only presented to professionals, owing to its complexity. e, environmental factor; GS, geriatric syndrome; ICF, International Classification of Functioning, Disability, and Health; pf, personal factor.

**Table 2:** The options included in geriatric syndromes/conditions and personal factors.

Questions	Items
A1. Geriatric problems or concepts (Geriatric syndromes/conditions)	Social network, social support, social isolation, social participation, social role, life space, homebound, frailty, polypharmacy, and non-adherence
B4. Personal factors *	Sex, race, sex, age, strength, lifestyle, cultural customs, coping method, social background, education, occupation, personality, experience, and individual’s psychological characteristics

* As pfs are ICF components without specific categories, representative items were proposed instead by the core members and presented to the respondents. ICF, International Classification of Functioning, Disability, and Health; pf, personal factor.

**Table 3:** The demographic characteristics of the respondents.

	Round 1 *n* = 327	Round 2 *n* = 182
Male/Female	168/159	90/90 ^1^
Healthcare professionals	80 (24.4%)	53 (29.1%)
Geriatricians	17	7
Nurses	23	22
Therapists	33	20
Case managers	7	4
Non-professional older adults	247 (75.6%)	129 (70.9%)
Registered respondents	242	126
Convenience sampling	5	3

^1^ Sex and age are missing for two respondents.

**Table 4:** Selected ICF components included in the classical CGA.

ICF Components	Selected ICF Categories
Body function (b) ^1^	b140 (attention functions), b144 (memory functions), b152 (emotional functions), b164 (higher-level cognitive functions), b110 (consciousness functions), b114 (orientation functions), b117 (intellectual functions), b122 (lobal psychosocial functions), b134 (sleep functions), b210 (seeing functions), b230 (hearing functions), b280 (sensation of pain), b410 (heart functions), b435 (immunological system functions), b440 (respiration functions), b455 (exercise tolerance functions), b510 (ingestion functions), b525 (defecation functions), b620 (urination functions), b710 (mobility of joint functions), b730 (muscle power functions)
Body structure (s) ^1^	s110 (structure of brain), s750 (structure of lower extremity)
Activities and participation (d)	d310 (communicating with—receiving spoken messages), d330 (speaking), d350 (conversation), d450 (walking), d470 (using transportation), d510 (washing oneself), d520 (caring for body parts), d530 (toileting), d540 (dressing), d550 (eating), d560 (drinking), d570 (looking after one’s health), d710 (basic interpersonal interactions), d760 (family relationships), d860 (basic economic transactions), d920 (recreations and leisure)
Environmental factors (e)	e110 (products or substances for personal consumption), e115 (products and technology for personal use in daily living), e120 (products and technology for personal indoor and outdoor mobility and transportation), e125 (products and technology for communication), e165 (assets), e230 (natural events), e310 (immediate family), e325 (acquaintances, peers, colleagues, neighbors, and community members), e355 (health professionals), e410 (individual attitudes of immediate family members), e575 (general social support services, systems and policies), e580 (health services, systems, and policies)
Personal factors (pf) ^2^	Sex, age, strength

^1^ Body function (b) and body structure (s) were presented to professional respondents only. ^2^ All items were selected from the presented list (see Table S2). CGA, comprehensive geriatric assessment; ICF, International Classification of Functioning, Disability, and Health.

**Table 5:** Answers to open-ended questions on states to be evaluated via the CGA *.

Classification by ICF Linking Rules 2016	Answers ^1^
nd	Adverse drug reactions
nd-hc	Health status
nd-dis	Falls, concomitant chronic diseases, hospital dependence
pf	Proactiveness
nc	Advanced care planning
nc-hc	Dementia, depression, sarcopenia, malnutrition, self-neglect
nc-QOL	QOL, life satisfaction, self-perception of social competence, Ikigai (the individual’s sense of meaning in life), perception of health status ^2^, self-evaluation ^2^, enjoying solitude ^2^, individual’s wishes ^2^, self-efficacy ^2^

^1^ Answers were restricted to responses that cannot be assigned to the second level of the ICF category codes. ^2^ Underlined answers are responses provided by non-professional respondents. CGA, comprehensive geriatric assessment; ICF, International Classification of Functioning, Disability, and Health; QOL, quality of life; nc, not covered; nd, not defined; pf, personal factor. * See Table S4 for a comparative example of ICF linking of SF-12 for reference.

**Table 6:** Summary of responses to question A1 (percentages of the selected items).

Items	Professional Respondents		Non-Professional Respondents
	Medical Staff *	Case Manager	
Social network	38 (77.6)	2 (50.0)	59 (45.7)
Social support	47 (95.9)	3 (75.0)	76 (58.9)
Social isolation	21 (42.9)	2 (50.0)	37 (28.7)
Social participation	45 (91.8)	3 (75.0)	76 (58.9)
Social role	35 (71.4)	2 (50.0)	49 (38.0)
Life space	34 (69.4)	1 (25.0)	63 (48.8)
Homebound	31 (63.3)	1 (25.0)	45 (34.9)
Frailty	35 (71.4)	1 (25.0)	26 (20.2)
Polypharmacy	27 (55.1)	0 (0)	40 (31.0)
Non-adherence	19 (38.8)	4 (25.0)	28 (21.7)

* The term “Medical staff” refers to certified geriatricians, nurse practitioners, and therapists.

**Table 7:** Comparison of items not having second-level ICF category codes.

Assessment Set	Items
Classical CGA	pf (proactiveness), nd (adverse drug reactions), nc (advance care planning), nc-QOL (self-perception), nd-hc (health status), nc-hc, and nd-dis (geriatric syndromes and concomitant chronic disease)
ICF Core Set	-
Frailty phenotype	-
Kihon Check List	hc (physical strength), nc (house-boundness)
GFI	nc-hc (dementia), nd (number of medications)
FI-CGA	pf (social), nc-hc (comorbidity scale), hc (falls)
CGA-Frailty	nc-hc (comorbidity scale)
70-item Frailty index	pf (family history), nc-hc (medical histories of some chronic disease), nc (other medical histories), nd (changes in everyday activities)
G8	pf (age, health status), hc (number of neuropsychological problems), nc (number of medications)
VES-13	pf (age, self-reported health)

Items without second-level ICF category codes were obtained from reference [15]. Underlined items are not the answers directly answered. They are items summarized by the core members. CGA, comprehensive geriatric assessment; ICF, International Classification of Functioning, Disability, and Health; nc, not covered; nd, not defined; pf, personal factor; QOL, quality of life.

## Data Availability

Data presented in this study are available on request from the corresponding author. The data are not publicly available on request due to restrictions of the ethical committee.

## Supplementary Materials

The following supporting information can be downloaded at https://www.mdpi.com/article/10.3390/jcm12154917/s1, Figure S1: The structure of the ICF as described by the WHO; Figure S2. ICF linking decision tree for ICF linking rules 2016 (from reference [13] Figure 1 (Linking decision tree)); Table S1: ICF linking rules of refined 2016 version. (Reference [13] Table 1 modified); Table S2: Examples of ICF linking according to 2016 rules (Short-Form-12 and aims of other technical and clinical measures). References [5,13] are cited in Supplementary Materials.
