# Supplementary material for: Detecting Comparative Features of Comprehensive Geriatric Assessment through the International Classification of Functioning, Disability, and Health Linkage: A Web-Based Survey
Source: J Clin Med. 2023 Jul 26;12(15):4917. doi: 10.3390/jcm12154917 (PMC10419489; doi:10.3390/jcm12154917)
Supplement: Supplementary file 1 [file jcm-12-04917-s001.zip › jcm-2417996-supplementary.pdf]

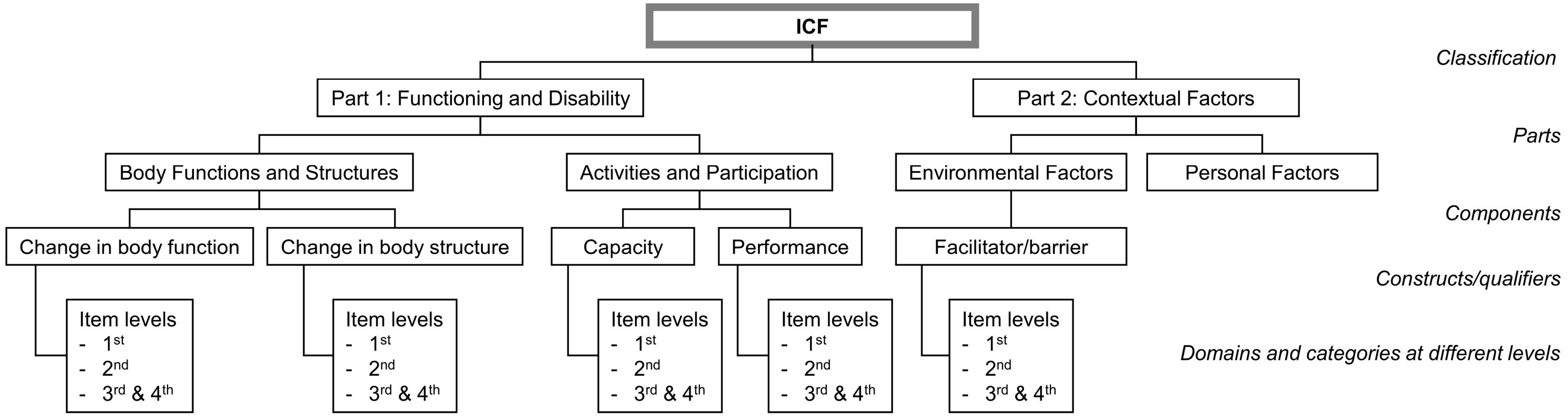

Figure S1. The structure of the ICF as described by the WHO [5]. The hierarchical ICF provides a unified and standard language and framework for describing health and health-related states and prompting the systematic coding of health components. ICF, International Classification of Functioning, Disability, and Health; WHO, World Health Organization.

Table S1. ICF linking rules of refined 2016 version. (Reference [13] Table 1 modified).

Rule	ICF linking refined rules (2016)
1	**【Understanding ICF】e.g. chapters, domains, categories**<br>Acquire good knowledge of the conceptual and taxonomical fundamentals of the ICF, as well as of the chapters, domains and categories of the detailed classification, including definitions before starting to link meaningful concepts to the ICF categories
2*	**【Identify overall scope (purpose) of the instrument before linking】**<br>Identify the purpose of the information to be linked by answering the question What is this piece of information about? Or What is this item about? The answer to these questions will help to identify the main concept(s) most relevant to be linked to the ICF
3	Identify any additional concepts contained in the piece of information in addition to the main concept(s) already identified in the previous step
4*	**【Identify perspectives】e.g. Performance, Capacity, Appraisal, Need, Dependency**<br>Identify and document the perspective taken on within a certain piece of information when linking it to the ICF
5**	【Categorize response options】**e.g. Intensity, Frequency, Duration, Agreement, Qualitative attributes**<br>Identify and document the categorization of the response options
6	【Identify the meaningful concepts and link to the ICF】<br>Link all meaningful concepts, the most relevant and additional ones, to the most precise ICF category
7*	**【Additional information for not specified or covered concepts <u>within the ICF category</u>】**<br>Use ''other specified'' or ''unspecified'' ICF categories as appropriate
8	**【Concepts not defined in ICF】e.g. "I have unpleasant side effects from my medication": (Main concept) side effect, (Most precise ICF category) nd**<br>If the information provided by the meaningful concept is not sufficient for making a decision about the most precise ICF category, assign the concept to nd (not definable)
9	【Concepts of **Personal factor】e.g. "… Your faith in God?": (Main concept) faith in God, (Most precise ICF category) pf**<br>If the meaningful concept is not contained in the ICF, but is clearly a personal factor as defined in the ICF, assign the meaningful concept to pf (personal factors)
10	**【Concepts not covered by ICF】e.g. "How would you rate your quality of life": (Main concept) quality of life, (Most precise ICF category) nc-qol**<br>If the meaningful concept is not contained in the ICF, assign this meaningful concept to nc (not covered)

* refined rules from that of 2005.

** The rule 5 is only relevant for questionnaires, assessments, or tests that contain response options.

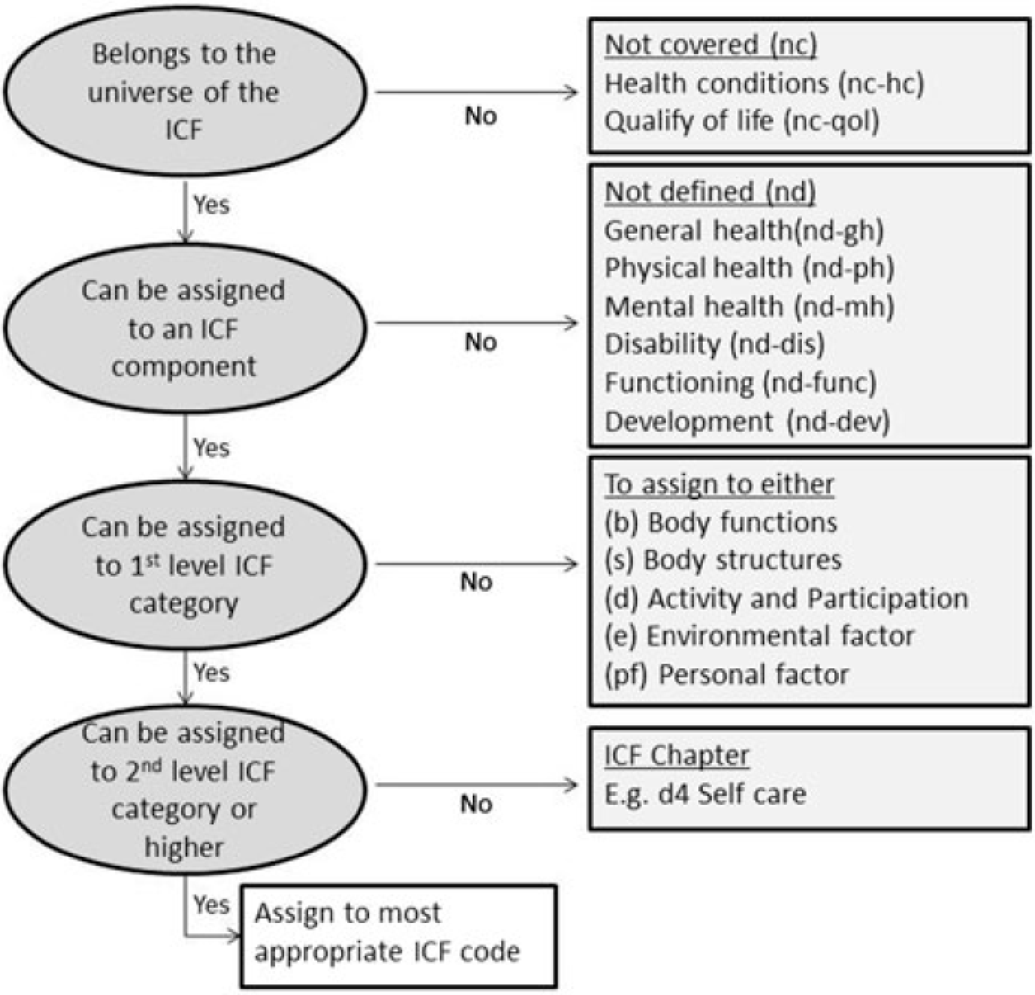

Figure S2. ICF linking decision tree for ICF linking rules 2016 (Figure 1 from reference [13] [Linking decision tree]).

Table S2. Examples of ICF linking according to 2016 rules (Short-Form-12 and aims of other technical and clinical measures).

	Classifications	Aims of technical and clinical measures [1]	Items of SF-12 questionnaires [2]
ICF items	b	Blood pressure, exercise tolerance, sleep, pain	6, 7, 8, 9, 10, 11, 12
	s	Bone structure, joint structure, prevention of skin ulcer	
	d	Walking	2, 3, 6, 7, 8, 12
	e		
	**pf**		
**Non-ICF items**	**nd**	**Liver function, Prevention of falls**	
	nd-gh		1, 2
	**nd-hc**		
	nd-ph		4, 5, 12
	**nd-dis**		
	**nc**	**Osteoporosis severity**	
	**nc-hc**	**Alcohol abuse, symptom severity**	
	**nc-QOL**		

The bolded non-ICF classifications were those included in classical CGA.

1 The data derives from Table IV of the reference [13]

2 The data derives from Table III of the reference [13].

Questionnaires of SF-12 are briefly summarized below:

Item 1. In general, would you say your health is (excellent, very good, good, fair, poor)

Does your health now limit you in these activities? If so, how much?

Item 2. Moderate activities, such as moving a table (1), pushing a vacuum cleaner (2), bowling, or playing golf (3): (Limited a lot, Limited a little, Not limited at all)

Item 3. Climbing several flights of stairs (Limited a lot, Limited a little, Not limited at all)

Have you had any of the following problems with your work or other regular activities s AS A RESULT OF YOUR PHYSICAL HEALTH?

Item 4. Accomplished less than you would like (Yes, No)

Item 5. Were limited in the kind of work or other activities (Yes, No)

Were you limited in the kind of work you do or other regular activities AS A RESULT OF ANY EMOTIONAL PROBLEMS (such as feeling depressed or anxious)?

Item 6. Accomplished less than you would like (Yes, No)

Item 7. Didn't do work or other activities as CAREFULLY as usual (Yes, No)

Item 8. How much did PAIN interfere with your normal work (including both work and outside) (Not at all, a little bit, moderate, quite a bit, extremely)

For each question, please give the one answer that comes closest to the way you have been feeling.

Item 9. Have you felt calm and peaceful (All of the time, Most of the time, A good bit of the time, Some of the time, A little of the time, None of the time)

Item 10. Did you have a lot of energy? (All of the time, Most of the time, A good bit of the time, Some of the time, A little of the time, None of the time)

Item 11. Have you felt downhearted and blue? (All of the time, Most of the time, A good bit of the time, Some of the time, A little of the time, None of the time)

Item 12. how much of the time has your PHYSICAL HEALTH OR EMOTIONAL PROBLEMS interfered with your social activities? (All of the time, Most of the time, A good bit of the time, Some of the time, A little of the time, None of the time)
